# Application of machine learning in predicting oil rate decline for Bakken shale oil wells

**DOI:** 10.1038/s41598-022-20401-6

**Published:** 2022-09-28

**Authors:** Subhrajyoti Bhattacharyya, Aditya Vyas

**Affiliations:** Deysarkar Centre of Excellence in Petroleum Engineering, Indian Institute of Technology Kharagpur, Kharagpur, West Bengal India

**Keywords:** Energy science and technology, Engineering, Mathematics and computing

## Abstract

Commercial reservoir simulators are required to solve discretized mass-balance equations. When the reservoir becomes heterogeneous and complex, more grid blocks can be used, which requires detailed and accurate reservoir information, for e.g. porosity, permeability, and other parameters that are not always available in the field. Predicting the EUR (Estimated Ultimate Recovery) and rate decline for a single well can therefore take hours or days, making them computationally expensive and time-consuming. In contrast, decline curve models are a simpler and speedier option because they only require a few variables in the equation that can be easily gathered from the wells' current data. The well data for this study was gathered from the Montana Board of Oil and Gas Conservation's publicly accessible databases. The SEDM (Stretched Exponential Decline Model) decline curve equation variables specifically designed for unconventional reservoirs variables were correlated to the predictor parameters in a random oil field well data set. The study examined the relative influences of several well parameters. The study's novelty comes from developing an innovative machine learning (ML) (random forest (RF)) based model for fast rate-decline and EUR prediction in Bakken Shale oil wells. The successful application of this study relies highly on the availability of good quality and quantity of the dataset.

## Introduction

### Literature survey

The main objective of this study is to develop a ML based model that can be employed for the prediction of production rate decline for a large number of Bakken Shale wells in a very shorter period. This method will be much faster than the commercial reservoir simulators as it does not require solving a large number of finite difference equations. The production from unconventional shale oil and gas was started many years back in the USA. Since then, numerous exploration companies have collected the data of significant number of oil and gas wells drilled and produced from these reservoirs, resulting in a large amount of horizontal well data. This information is available in several publicly accessible website databases^1^. Various data analytics methods can be used to evaluate publicly available data to uncover underlying patterns and sweet spots in these reservoirs that could be beneficial for future horizontal well development^2–4^. The most extensively utilized method for projecting future production from shale oil wells is the projection of production decline curves^5^. Decline curve models are mathematical equations used to model existing well production data and predict a future well decline^1^. Developing an empirical model of the production rate decline from the well's early performance and extrapolating this pattern into the future can predict future production potential and EUR. The most commonly utilized production decline curve model is the Arps Hyperbolic Model. However, fitting the Arps Hyperbolic Model to production data from shale oil wells has frequently resulted in physically unrealistic values of hyperbolic decline coefficient^1^. SEDM was employed to predict production from unconventional wells to solve this challenge^5^. SEDM is better suitable for shale oil wells than Arps Hyperbolic Model, because they are in a transient flow regime during most of their lifetime. For positive ${q}_{i}$, n, and SEDM, SEDM returns a finite EUR value^1^. As a result, SEDM was used in the study to predict production rate decline and EUR for test wells.

In a similar study, an alternate approach for rate/pressure deconvolution was presented. The physics-based trained parameters and algorithms play a key role in effectively implementing the recommended strategy by preserving superposition transient flow physics^6^. The primary drawback of this study is that this method fails to give satisfactory results when very highly variable and limited data is available. The principal drawback of this study is that it is highly dependent on the availability of a sufficient quantity of data. Another study proposed a model for predicting the permeability of a technically challenging (extremely heterogeneous) carbonate rock premised on the Random Forest regression, which can acquire proficiently from the reliant physical parameters and provide an assured permeability prediction when compared with conventional empirical models^7^. The principal drawback of this study is that it is highly dependent on the availability of good quality of noise-free data. In a similar study, the authors employed data-driven modeling for predicting the rate decline of Eagle Ford Shale oil wells^8^. Another study proposed an ANN-based model for predicting the rate decline of Eagle ford Shale oil wells^9^. The primary drawback of these studies was that their applicability was restricted only to Eagle for shale oil wells.

In a similar study, Fuzzy logic, ANN (Artificial Neural Network), and Imperialist Competitive algorithms were compiled to build a model for the prediction of oil flow rate^10^. The main drawback in this study is the determination of the optimized ANN architecture. Another study compiled several machine learning algorithms to predict porosity and permeability through the inclusion of petro-physical logs^11^. The primary drawback of this study is the involvement of complicated machine learning algorithms that takes an excessive amount of time. In another study, the authors presented a deep belief network (DBN) model for predicting the production of unconventional wells reliably and accurately. The authors run 815 numerical simulation cases for developing a database for model training and optimizing the hyper parameters by employing the Bayesian optimization algorithm. The proposed modeling framework was able to predict the production of unconventional wells more reliably and accurately than as compared to traditional machine-learning techniques. The primary limitation of this study is that the model training requires a lot of simulation runs to be performed^12^.

### Research problem

Commercial reservoir simulators can take hours or even days to forecast rate decline for a single well^13–16^. Commercial reservoir simulators solve the discretized form of mass balance equations. The number of grid blocks used in a reservoir model might be in the millions, requiring solving million-by-million matrix equations. As the reservoir becomes increasingly heterogeneous and complicated, a finer resolution model (with a higher number of grid blocks) should be used. Additionally, precise and comprehensive reservoir parameters, including porosity, permeability, saturation, and other variables, are essential to execute one or even more reservoir simulations for the wells considered in the study, which are not always available in the field.

### Objective and novelty

An alternative method based on machine learning-based has been presented in this study which is very fast and accurate since it does not necessitate solving matrix-based equations. It makes predictions based on previously collected field data. Machine learning can be utilized as an efficient tool to predict oil rate decline in the type of data presented in this study. This study took less than a minute to estimate the rate decline for all the wells used for predictions. In machine learning-based predictions, it has been observed that using the entire dataset to develop a machine learning model can result in considerable errors owing to data variability. To overcome this limitation, an alternative approach used in this work, included Cross-Validation employing k-fold Validation and Model Averaging using the ensemble technique (Polyak–Ruppert averaging).This method splits training data into multiple folds (k-folds) or subsets of data points, and a model was evaluated in one of the folds while the other folds were used for training. As a result, by applying different subsets of training data to minimize the over fitting problem, we will have multiple machine learning models derived from a single training data set at the end of the training. The final prediction for test data/new data is based on a weighted average of predictions made by all these models.

In this study, the variable ranking was used to show which variables/parameters significantly impact rate decline prediction and to rank them in order of priority. This data analysis was carried out to understand the dataset before using it to make predictions. This study also employed exploratory analysis to incorporate human judgement for more accurate conclusions.

### Area of study

**Figure 1:**
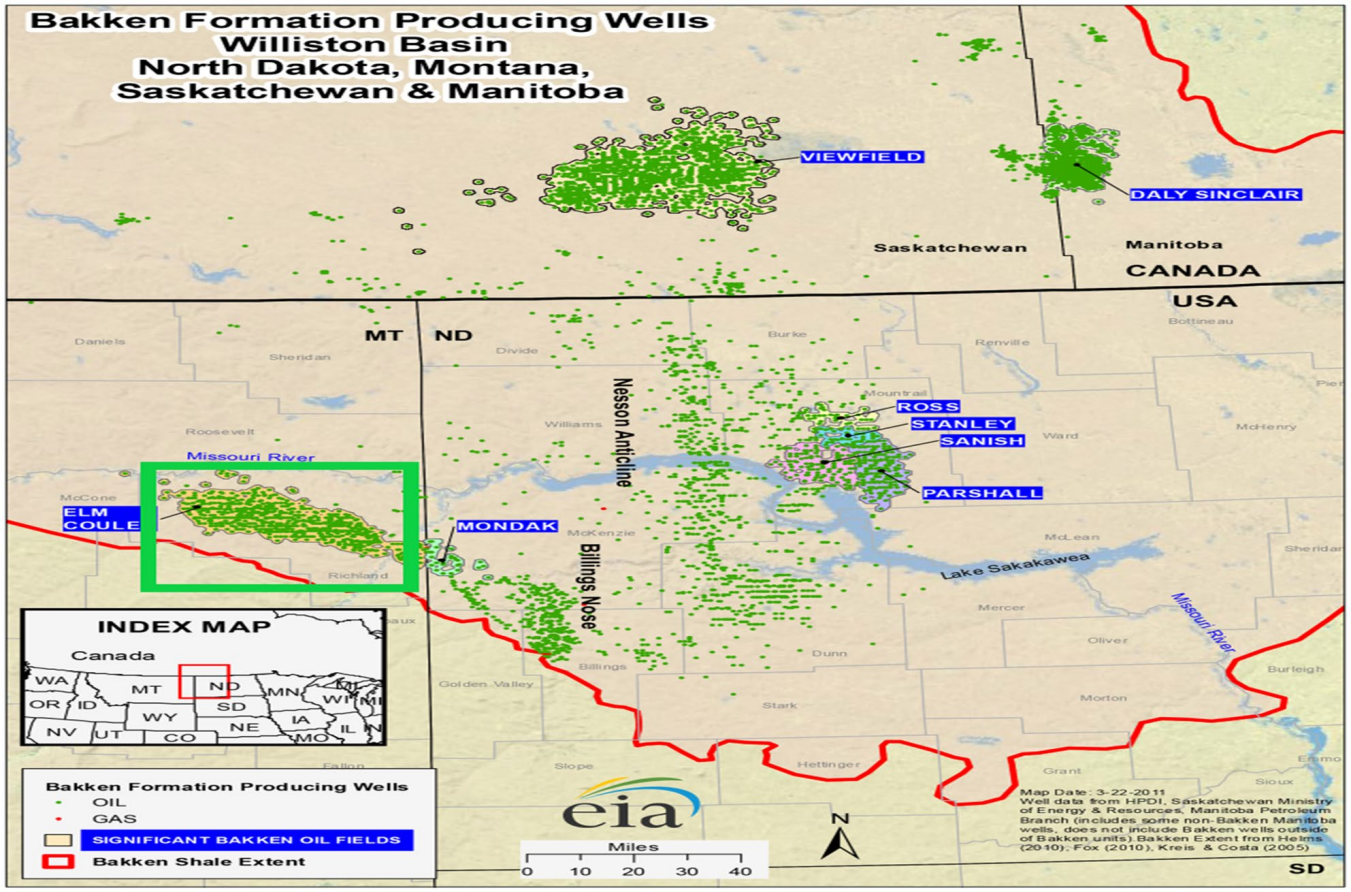
Bakken Shale region with oil and gas wells (natural gas intelligence)^17^.

North Dakota, South Dakota, Montana, Manitoba, and Saskatchewan are all part of the Williston Basin, which includes the Bakken Shale and its three forks. The Bakken shale can be seen in Fig. 1 with oil and gas wells (Natural Gas Intelligence). All of the oil wells in this study were selected from the Bakken Shale in Richland County (included in the green rectangle). SEDM was employed in this work to forecast production decline.

## Results and discussion

### Exploratory analysis

Figures 2, 3, 4, 5, 6, 7, 8, 9, 10, 11, 12, 13, 14, 15, 16, 17 and 18 depict the distribution of various parameters in the four clusters under investigation. They were solely employed for exploratory analysis, not for machine learning-based rate decline prediction. Figures 10, 11, 12, 13, 14 and 15 show the initial 24-h well potential test results. Figures 16, 17 and 18 give information related to hydraulic fracturing treatment.

**Figure 2:**
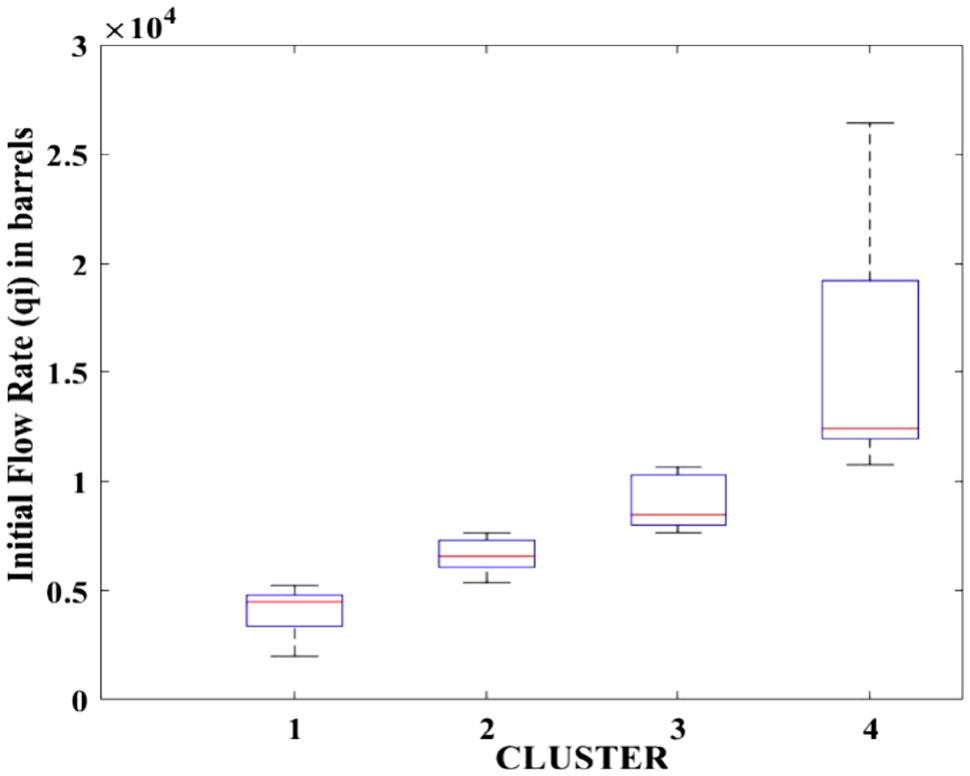
Initial flow rate $({q}_{i}).$

**Figure 3:**
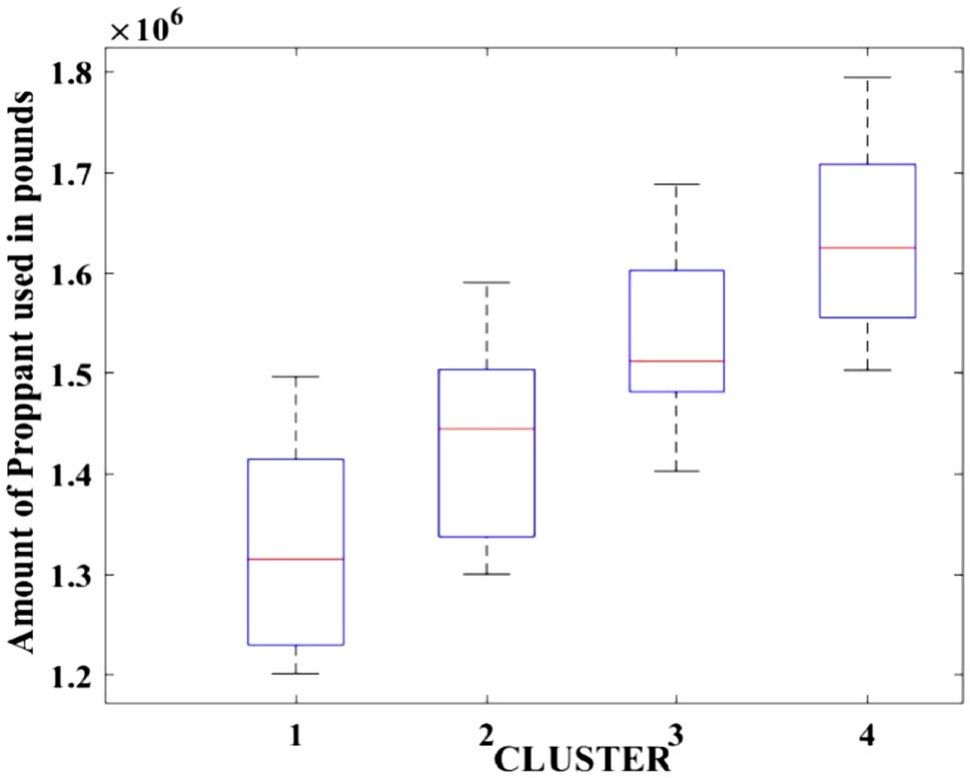
Amount of proppant used.

**Figure 4:**
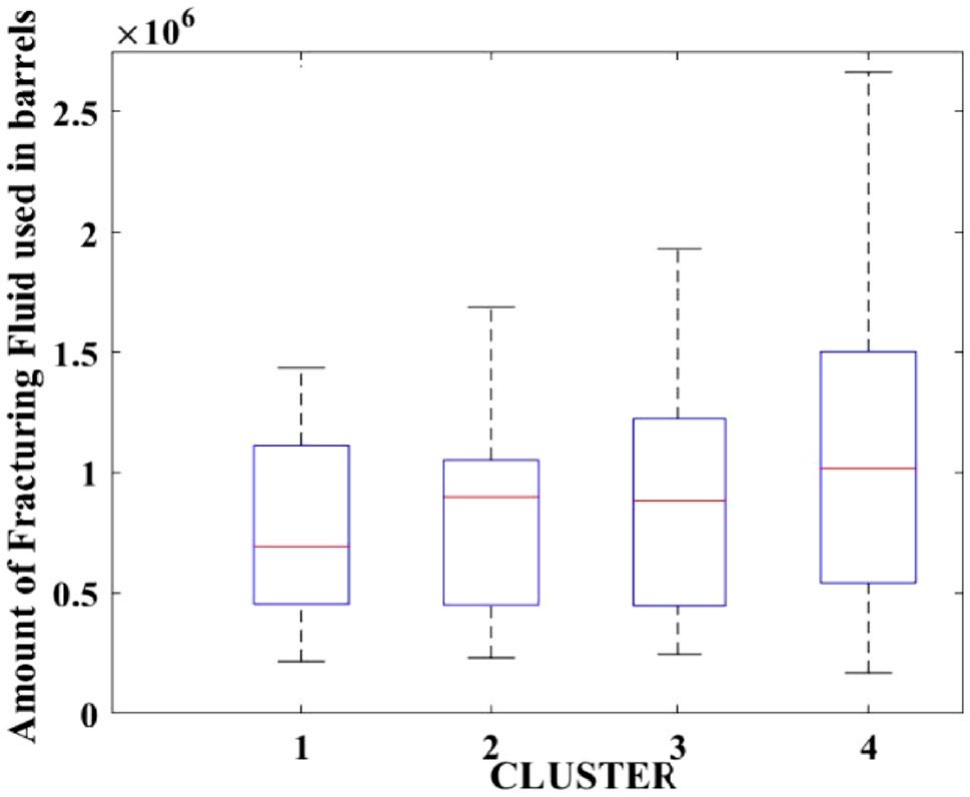
Amount of fracturing fluid used.

**Figure 5:**
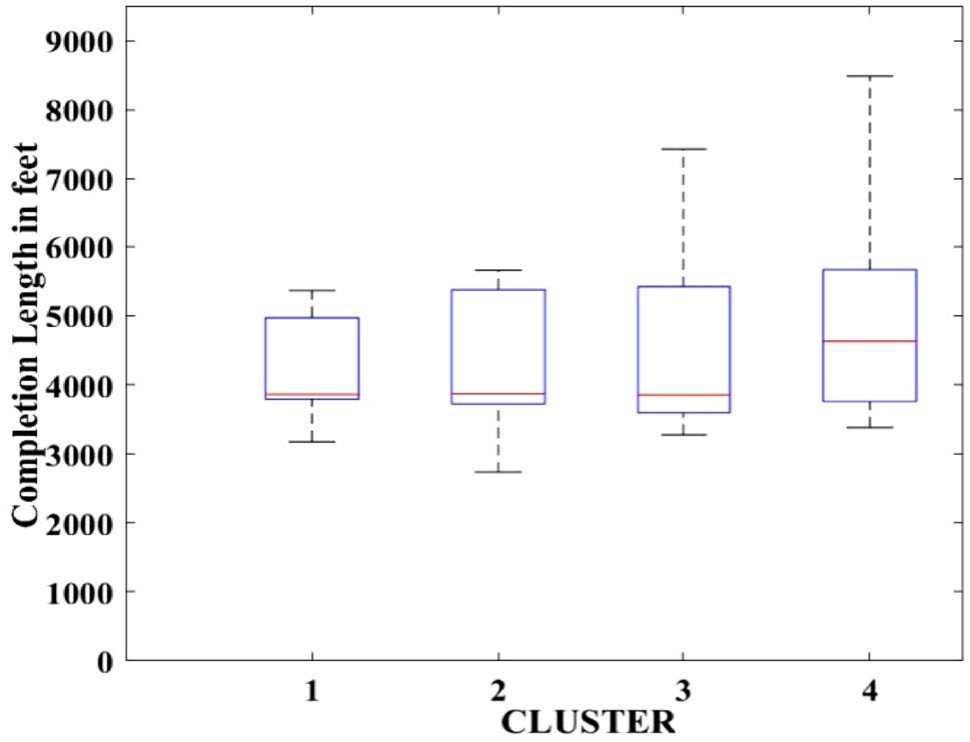
Completion length.

**Figure 6:**
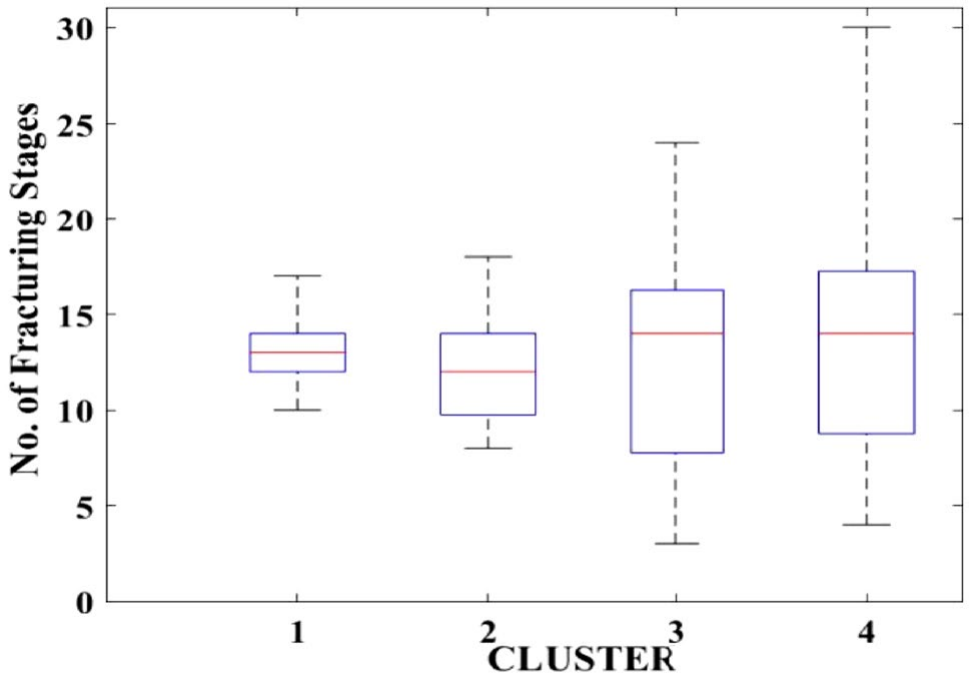
No. of fracturing stages.

**Figure 7:**
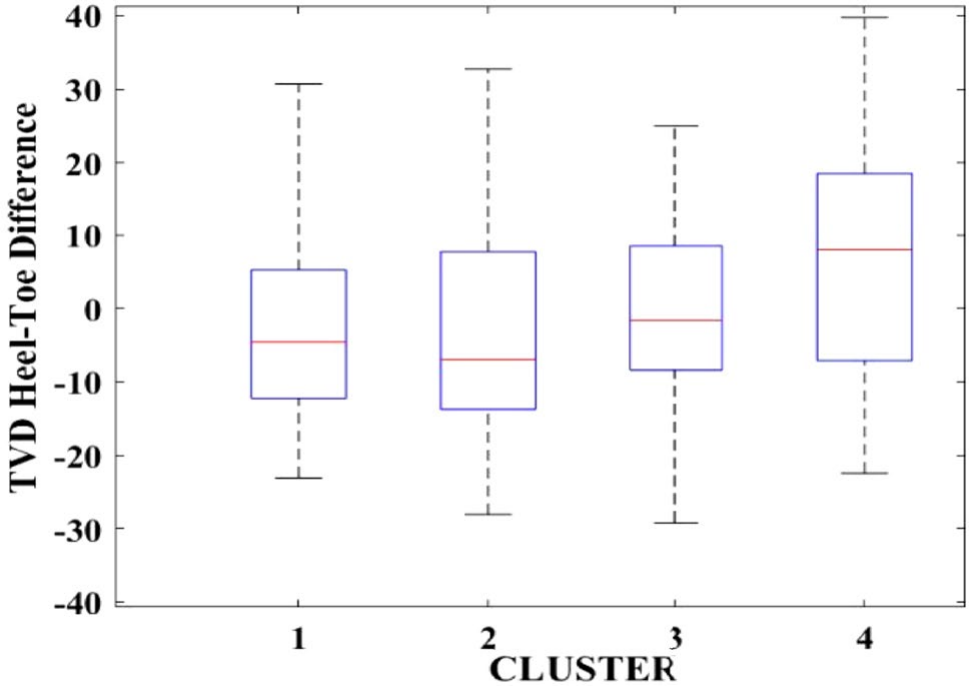
TVD heel-toe difference.

**Figure 8:**
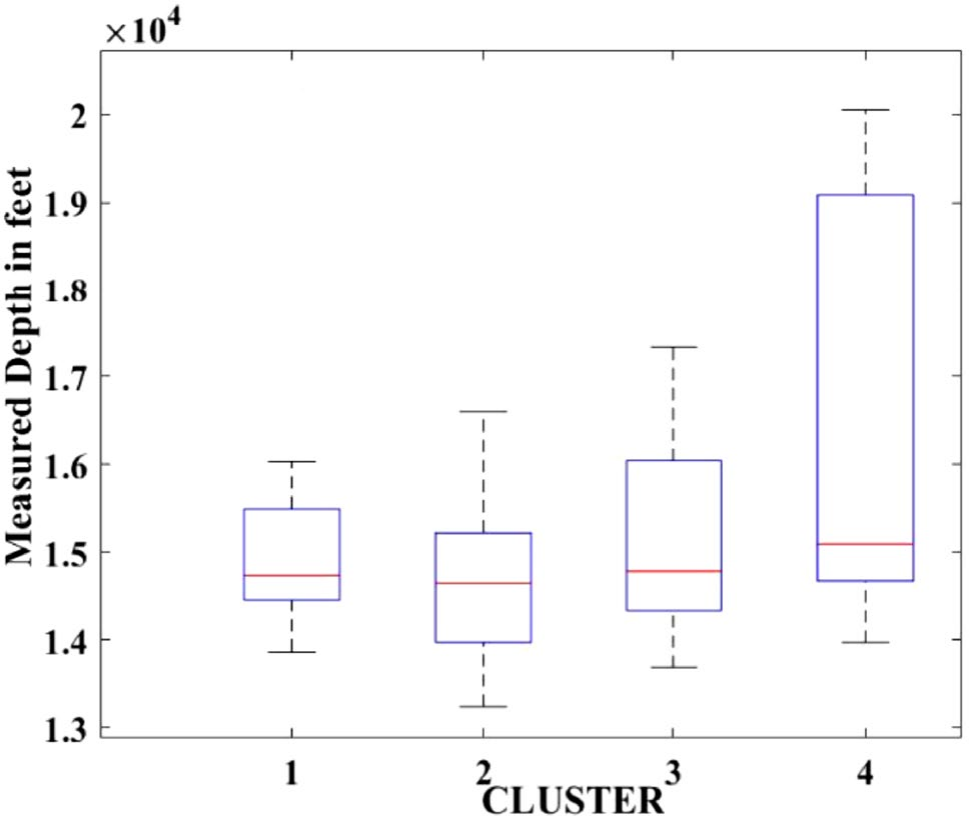
Measured depth.

**Figure 9:**
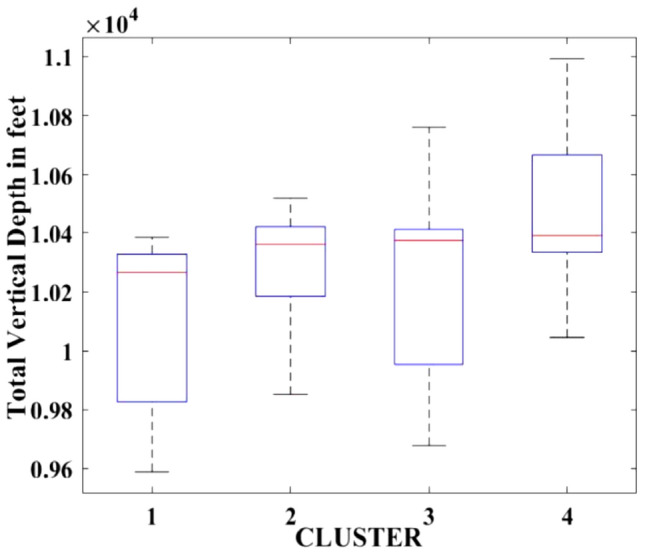
Total vertical depth.

**Figure 10:**
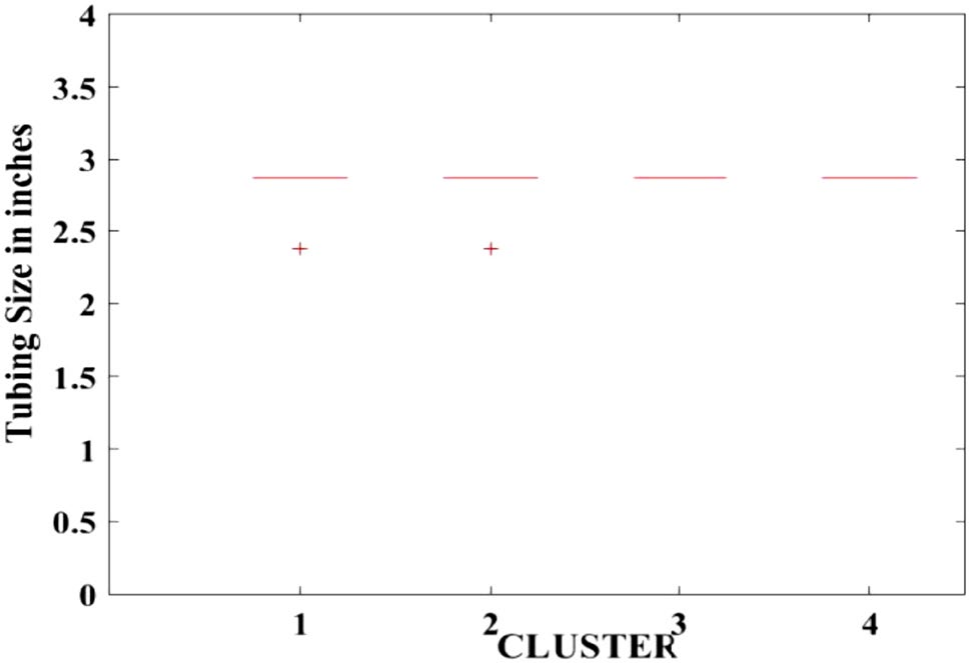
Intial-24 h period tubing size.

**Figure 11:**
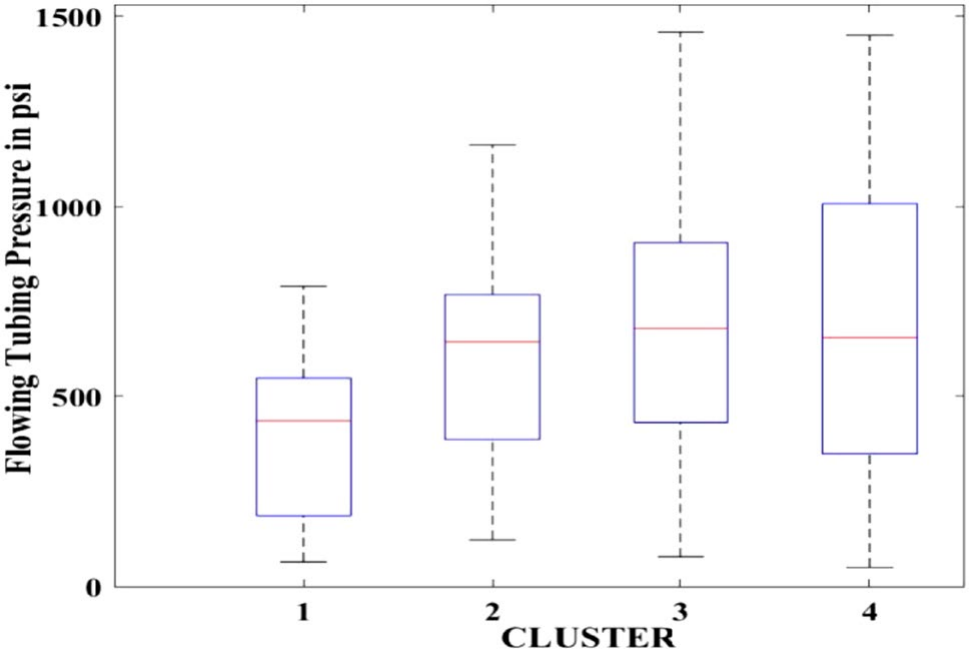
Initial 24-h period flowing tubing pressure.

**Figure 12:**
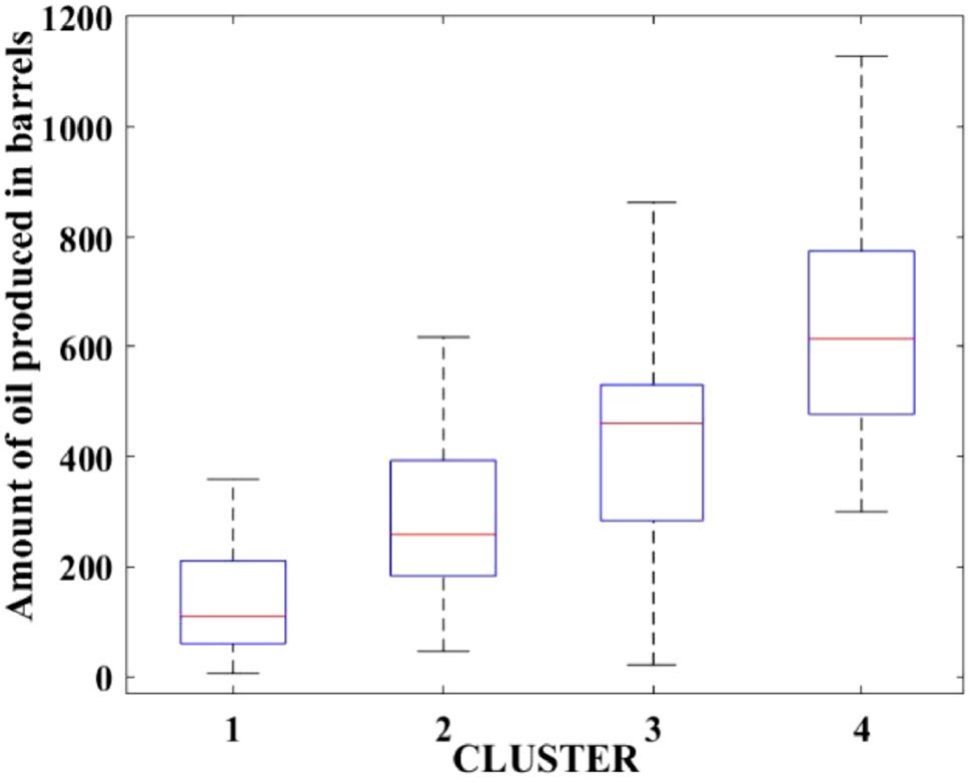
Initial 24-h period oil produced.

**Figure 13:**
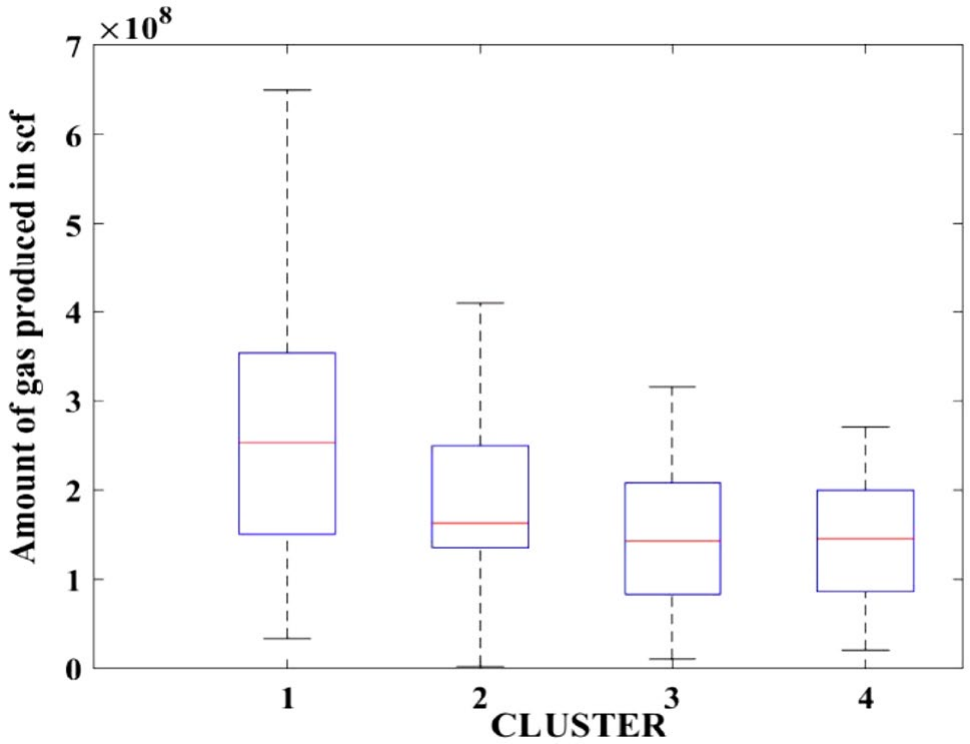
Initial 24-h period gas produced.

**Figure 14:**
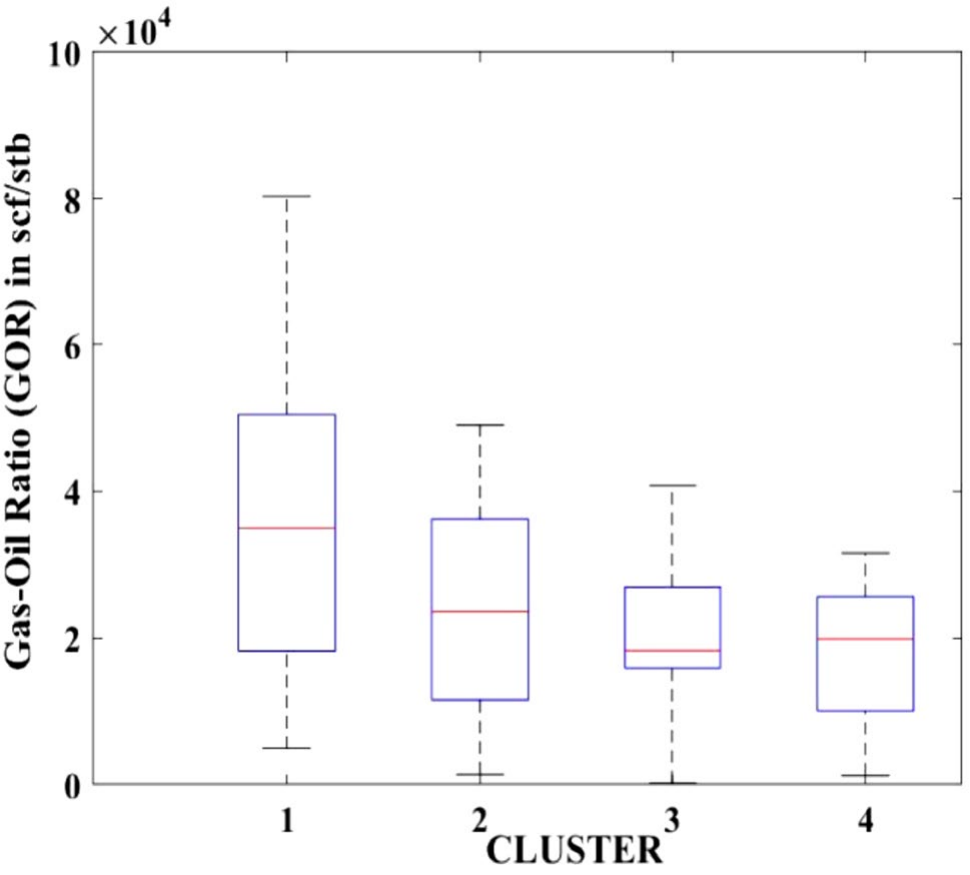
Initial 24-h period GOR.

**Figure 15:**
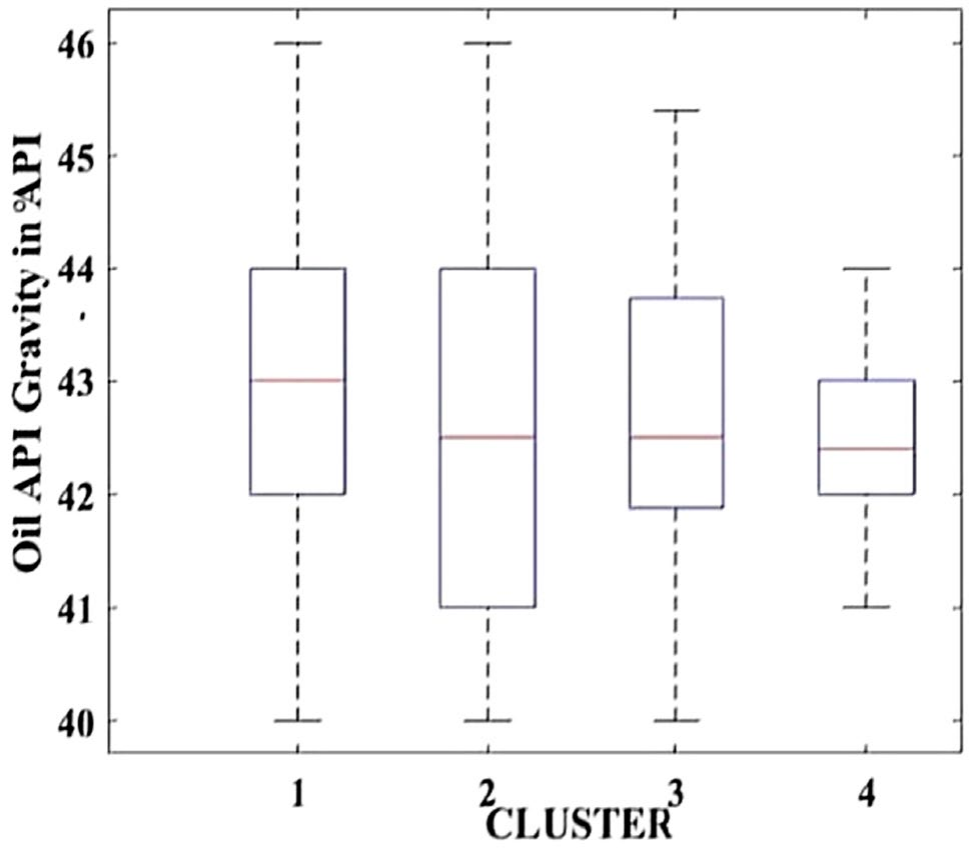
Initial 24-h period oil API gravity.

**Figure 16:**
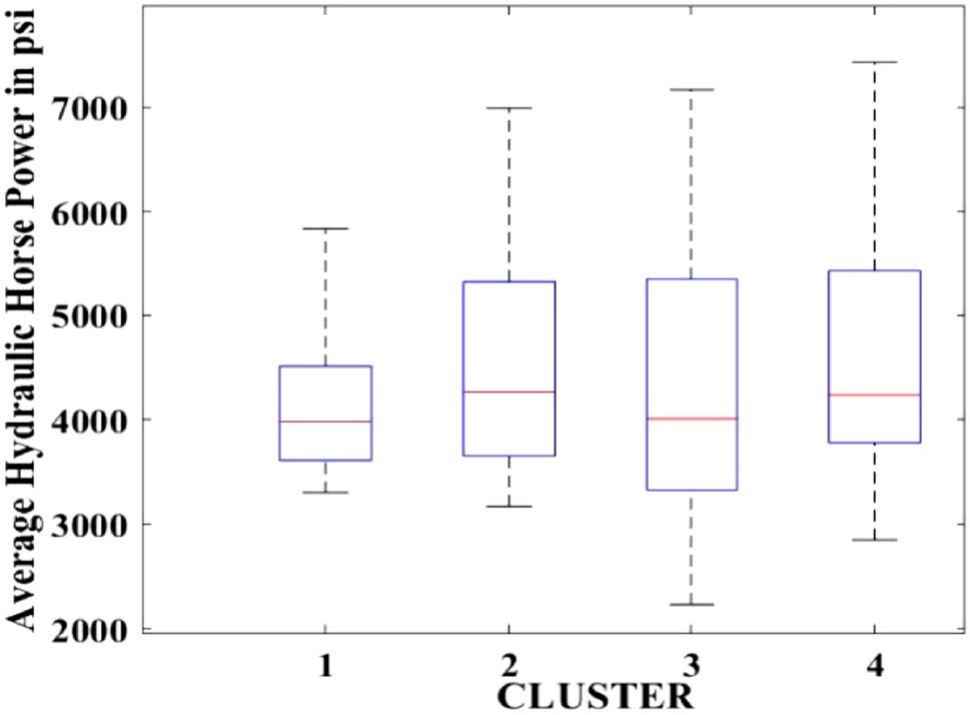
Average hydraulic horse power.

**Figure 17:**
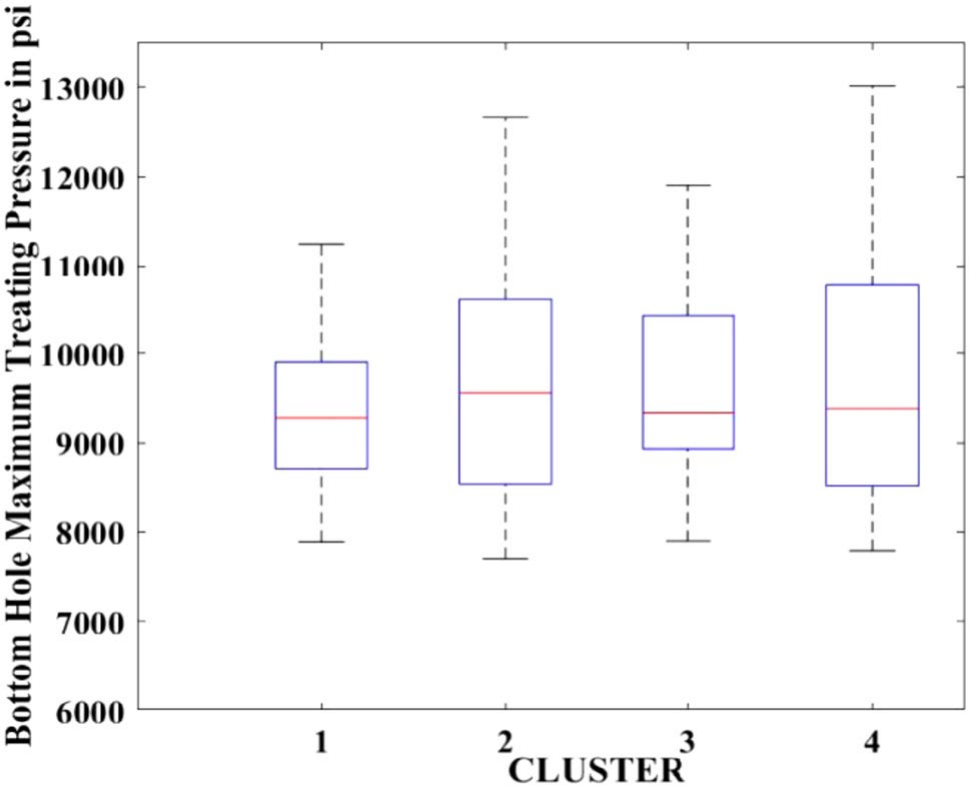
Bottom hole maximum treating pressure.

**Figure 18:**
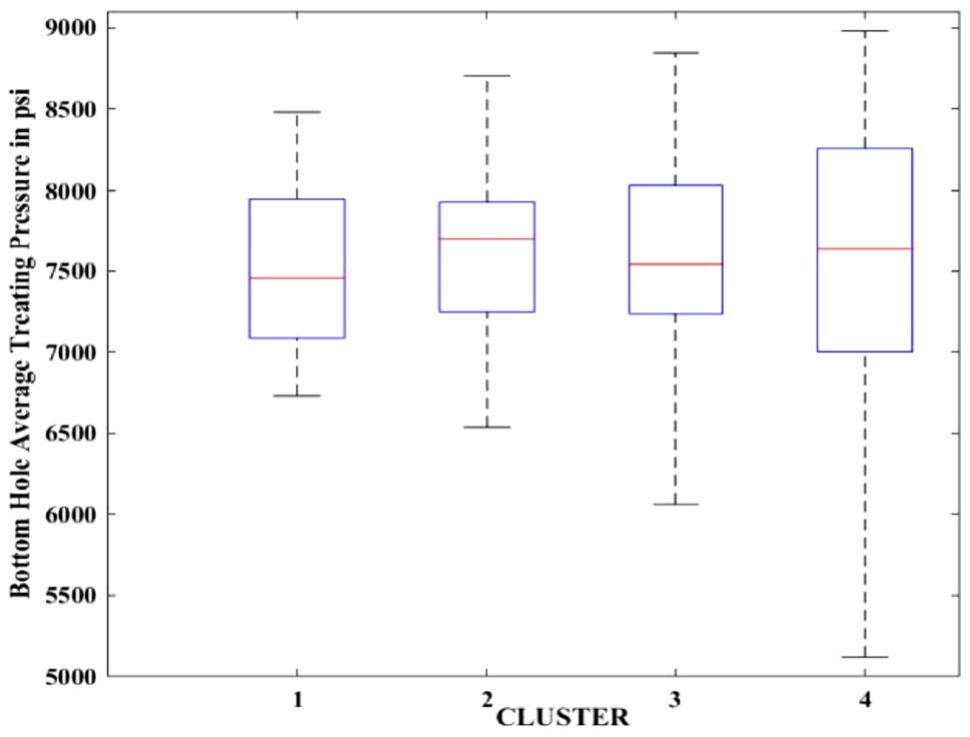
Bottom hole average treating pressure.

After dividing the well data into the four clusters, as shown in Figs. 2, 3, 4, 5, 6, 7, 8, 9, 10, 11, 12, 13, 14, 15, 16, 17 and 18, and comparing clusters nos. 1 and 4 wells, the following conclusions can be inferred:

- The median value and ${q}_{i}$ of cluster no. 4 wells are significantly higher than those of cluster no. 1 wells, as shown in Fig. 2. This is because the wells were clustered solely based on ${q}_{i}$.
- The proppant amount used in cluster no. 4 wells is higher than in cluster no. 1 wells, as shown in Fig. 3. There is a strong positive relationship between ${q}_{i}$ and proppant amount used.
- The median value and fracturing fluid amount employed in cluster no. 4 wells are much higher than in cluster no. 1 wells, as shown in Fig. 4. This is due to the fact that the proppant amount employed is related to the fracturing fluid amount.
- The median value and completion length used in cluster no. 4 wells are much higher than in cluster no. 1 wells, as shown in Fig. 5. This is because the proppant amount is related to the completed length.
- The range of values for the number of fracturing stages in cluster no. 4 wells is substantially more extensive than in cluster no. 1 wells, as shown in Fig. 6. This figure could not be used to derive any definitive conclusions.
- The median value and the TVD heel-toe difference in cluster no. 4 wells are comparable to those in cluster no. 1 wells, as shown in Fig. 7. The explanation is that all of the wells used in this study were from the same county. Considering the slight TVD heel-toe difference relative to the long completed lengths, the wells are quite close to horizontal.
- The upper limit of the range of measured depth values in cluster no. 4 wells is much higher than in cluster no. 1 wells, as shown in Fig. 8. In terms of completion length, a similar conclusion has been obtained.
- The total vertical depth in cluster no. 4 wells is considerably larger than in cluster no. 1 wells, as shown in Fig. 9. This shows that ${q}_{i}$. and TVD have a strong relationship.
- In the initial 24-h well potential test, only two tubing sizes were used for all of the selected wells, as shown in Fig. 10. As a result, the initial flow rate is relatively unaffected by tube size.
- The median value and tubing pressure in cluster no. 4 wells are higher than those in cluster no. 1 wells, as shown in Fig. 11. Cluster no. 4 wells have larger initial flow rates than cluster no. 1 wells, as previously observed. This may be because cluster no. 4 wells used longer completion lengths and proppant amounts, resulting in increased reservoir pressure during production.
- Figs. 12, 13, and 14 demonstrate that the initial 24-h period of oil produced in cluster no. 4 wells is higher than in cluster no. 1 wells. In contrast, the initial 24-h period gas produced and initial 24-h period GOR are lower. This indicates that cluster no. 4 wells have lower gas content.
- The range of Oil API Gravity in clusters no. 1 and 4 wells, as shown in Fig. 15, is nearly the same, i.e., between 40 and 46. This implies that the oil quality is comparable, and based on normal API ranges, all of the selected wells produce light crude oil because their API ranges are more than ${31.1}^{\mathrm{o}}$.
- Figs. 16, 17, and 18 shows that a definitive conclusion could not be made by comparing them with clusters for initial flow rates. However, all these variables are included in the remaining part of the current study.

### SEDM model prediction

**Figure 19:**
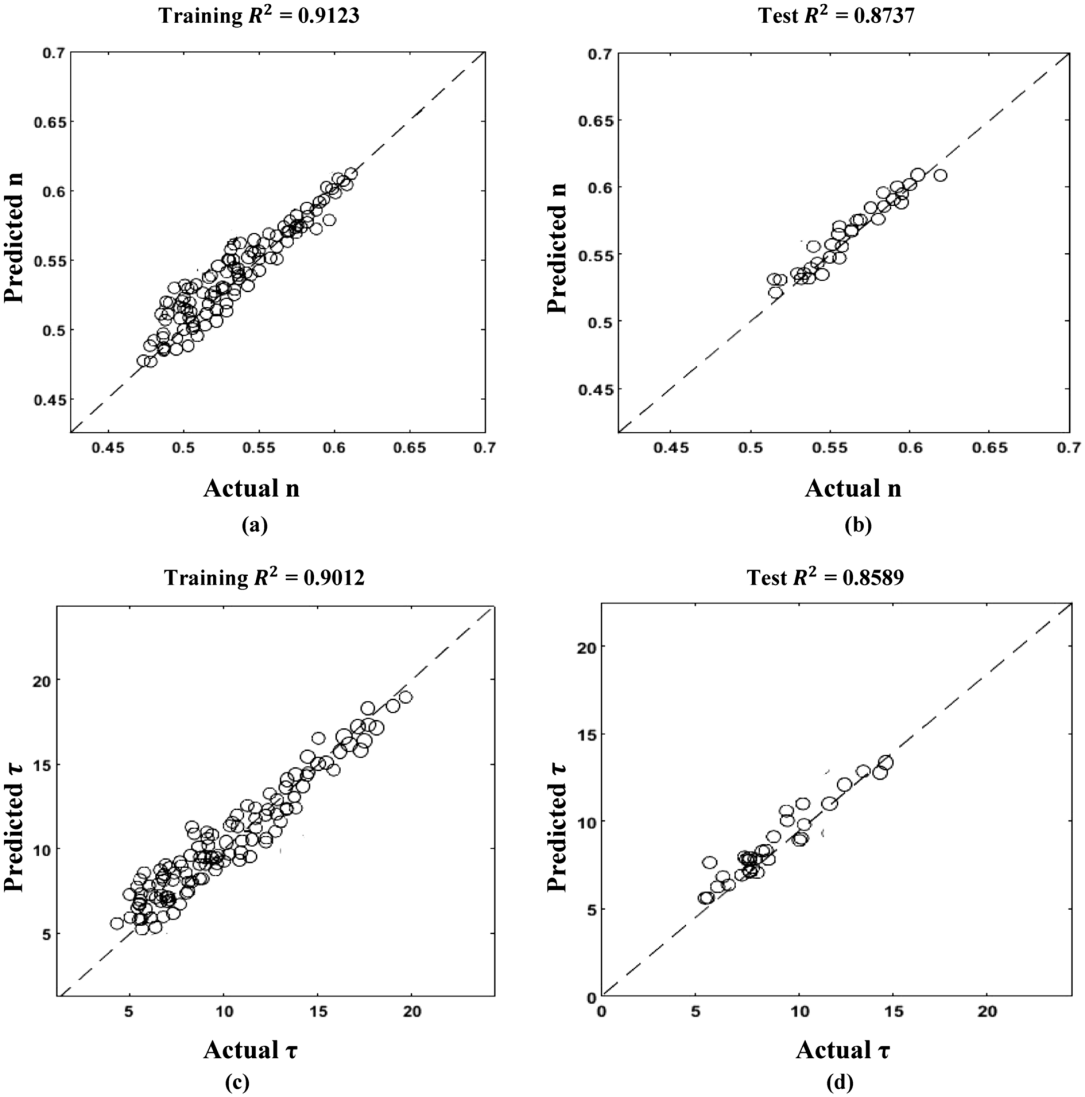
SEDM decline model parameter prediction.

**Figure 20:**
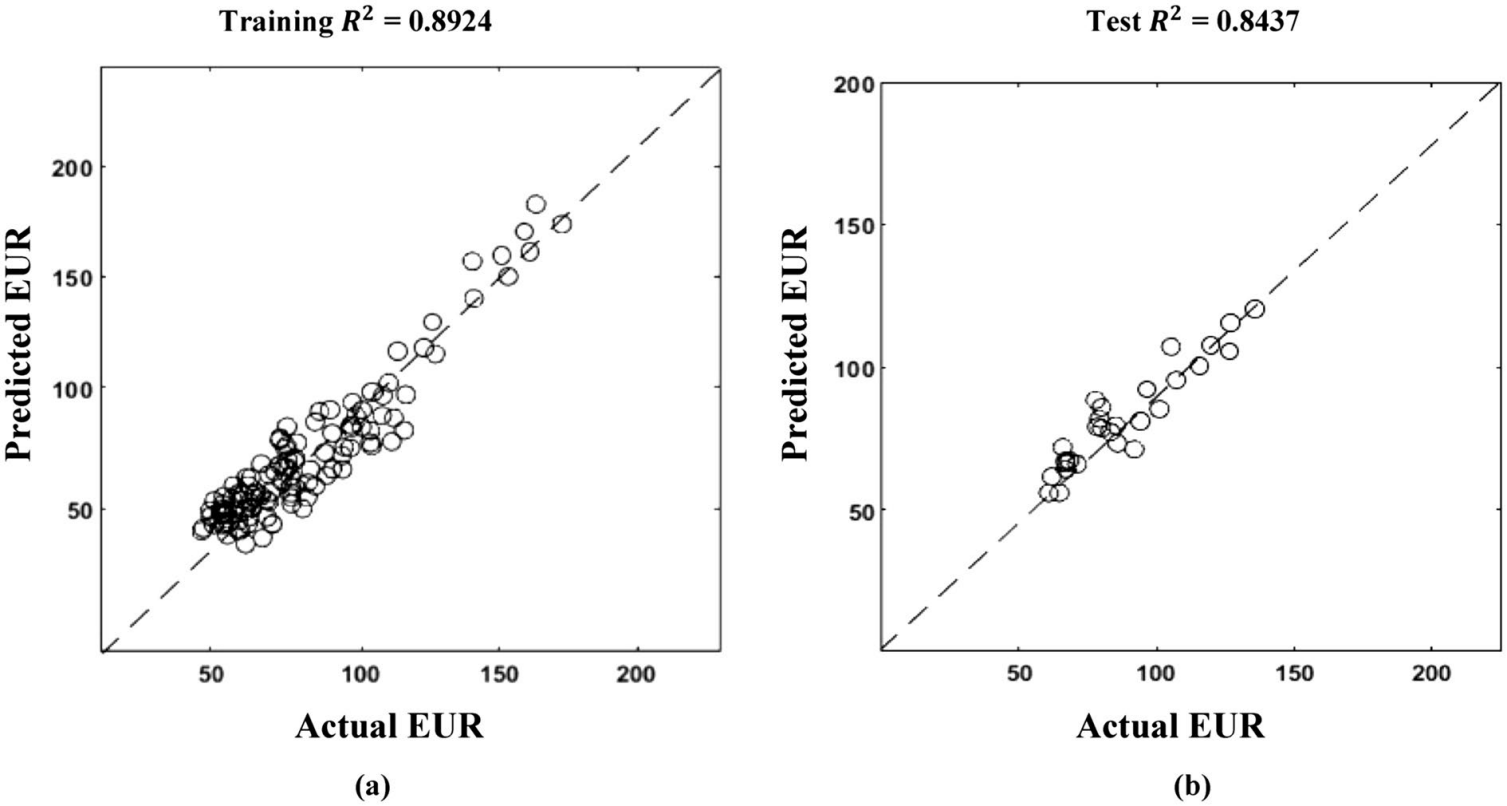
SEDM decline model EUR prediction.

**Figure 21:**
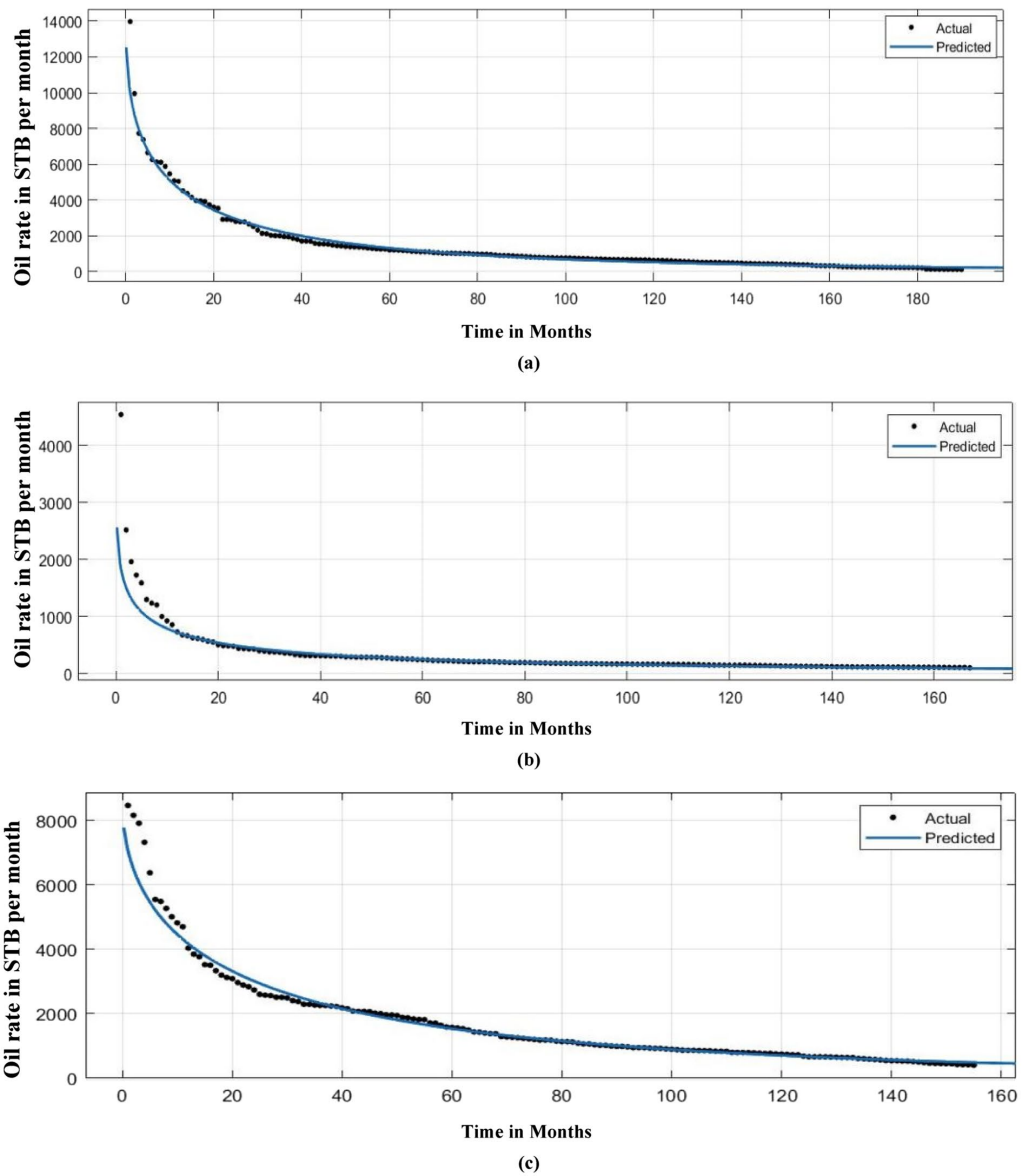

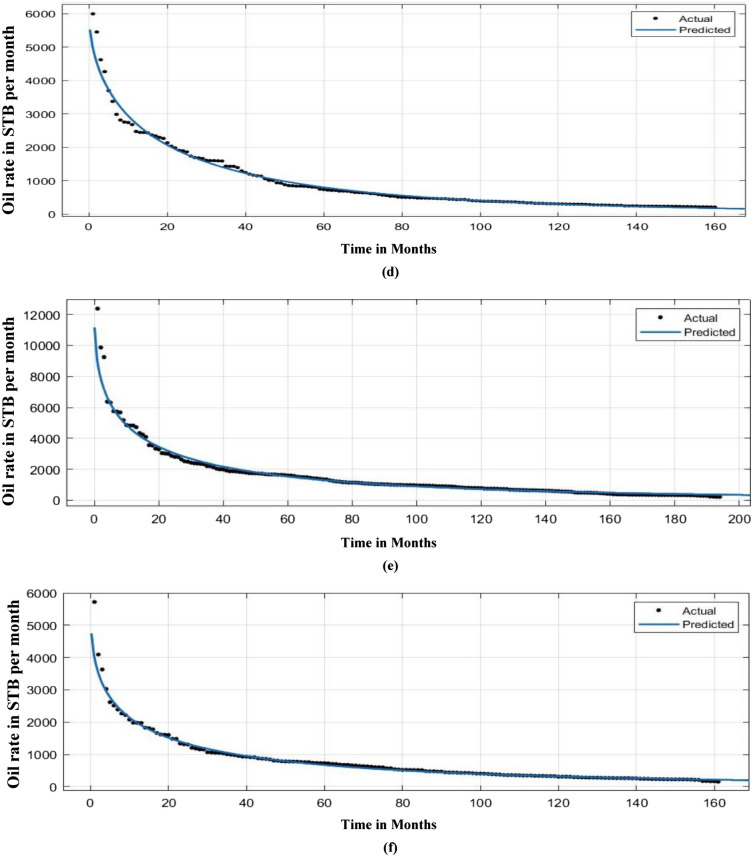
Fitting of SEDM decline model-based prediction for test wells.

Figure 19 describes the correlation between actual and predicted n and tau values. Figure 20 illustrates the correlation between EUR predicted and actual values. Following the prediction of decline variables for a test well, a decline curve for the well could be easily produced and matched to real production rate data. This is shown in Fig. 21 for several test wells using the machine learning model with the least test data root mean square errors (RMSE) for EUR.

In this study, we filtered out the wells with very high noise levels of production data and employed only those wells with smooth decline in production data. From Fig. 21a–f it can be observed that the production rate decline predicted by this methodology coincides more in the later part of the production period than compared to the earlier production period due to the presence of more level of noise in the early phase of production life.

### Variable ranking

The predictor variables were arranged in compliance with their numerical order priority using the Chi-Square test and F-test algorithm to determine which parameters can significantly affect rate decline, as shown in Table 1. A variable's median rank should be close to unity, and the rank variance should be low, if it is to be given more priority. It can also be employed for eliminating the features but due to the availability of limited data, the entire dataset is utilized, and none of the features were removed.

**Table 1:** Predictor variables ranking by the two Machine Learning Algorithms.

Predictor variables	Machine learning algorithm	
	Chi-square test	F-test
qi	1	1
Proppant amount used	2	3
Fracturing fluid used	3	2
Completion length	4	6
Flowing tubing pressure	5	7
Fracturing stages	6	4
Initial 24-h period gas–oil ratio (GOR)	7	5
Initial 24-h period gas produced	8	10
Measured depth	9	11
Total vertical depth	10	8
Initial 24-h period oil produced	11	9
Oil API gravity	12	14
Average hydraulic horse power	13	15
Tubing size	14	17
Bottom hole maximum treating pressure	15	12
Bottom hole average treating pressure	16	13
TVD heel toe difference	17	16

The predictors can be classified into three groups based on Table 1.

- **Most important predictors** ${q}_{i}$, proppant amount, fracturing fluid amount, completion length, tubing pressure, number of fracturing stages.
- **Moderately important predictors** Gas–oil ratio, amount of gas produced, measured depth, total vertical depth, amount of oil produced, oil API gravity.
- **Least important predictors** Hydraulic horse power, tubing size, bottom hole maximum treating pressure, bottom hole average treating pressure, TVD heel-toe difference.

Similar results in terms of predictor variable ranking^1^.

In this study, it was observed that ${q}_{i}$, proppant amount, and fracturing fluid amount are the most important predictors that influence the rate decline prediction of test wells which in turn also matches with the general concepts of fluid flow.

## Conclusions

1. Exploratory analysis reveals that the ${q}_{i}$ and proppant amount used in cluster no. 4 wells are much higher than those in cluster no. 1, suggesting the existence of a significant positive correlation between ${q}_{i}$ and amount of proppant utilized.
2. The predictor parameters were effectively correlated to SEDM decline curve parameters (n and τ) in a random collection of Bakken Shale oil well data utilizing machine learning. Test wells' oil flow rate decline curves were successfully predicted and matched to actual field data. Therefore, machine learning may be considered a reliable alternative to reservoir simulation.
3. This study employed exploratory analysis, machine learning modeling, and human judgment to draw better conclusions from the Bakken oil shale data.
4. The variable ranking shows that the ${q}_{i}$ is the most important predictor while the TVD Heel-Toe Difference is the least important predictor.
5. The primary reason for the TVD Heel-Toe Difference to be the least important predictor is the slight variation in its data as all the wells employed in this study were collected from the same county, i.e., all the wells were located near to each other.
6. In this study, decline curves have been extrapolated to large times, say 30 years and numerical integration has been employed to determine the actual EUR values. Since EUR is calculated based on qi and qi is the most important predictor that affects EUR. So, there will be a strong correlation between the two.

## Stretched exponential decline model (SEDM)^5^

The hyperbolic model had specific difficulties correlated with reserve forecasts for the long term. To overcome this, Valko formulated the SEDM, in which the production rate declines with time, as shown in Eq. (1):

1 $$ {\text{q}}\left( {\text{t}} \right) = {\text{q}}_{{\text{i}}} exp\left[ { - \left( {{\text{t}}/\uptau } \right)^{{\text{n}}} } \right] $$

where, q(t)  is the rate at a time t, *STB*/month, *q*_*i*_ is the initial rate, *STB*/month, τ is the characteristic relaxation time, months, *n* is the exponent parameter, dimensionless, *t* is the time, months.

“Compared to the Arps formalization, the new approach offers numerous advantages; among them the two most significant ones are the bounded nature of EUR from any individual well and the straight-line behavior of the recovery potential expression versus the cumulative production. For positive *n, τ* and ${q}_{o}$, the model gives a finite value of the EUR, even if no cutoff is used in time or in rate. (Unfortunately, the Arps family of curves leads to an unbounded and physically impossible estimate of EUR for *b* ≥ 1). Once the *n* and ${q}_{o}$ parameters are determined, a straight line plot of recovery potential vs cumulative production can be constructed from rates and the EUR can be read as the x-intercept. (For the Arps model family, the concept of recovery potential cannot even be defined for *b* ≥ 1)”^5^.Therefore, only SEDM was employed in this study.

## Machine learning algorithms

This study employed RF for test wells to forecast SEDM model parameters, decline curves, and EUR, as it is one of the most extensively used machine learning algorithms. It gave excellent prediction results as presented in previously published literature^1^.

A short description of Random Forest (RF) is given below:

### Random forest (RF)^1^

A Random Forest is a machine learning approach that consists of many uncorrelated trees (classification or regression trees), each modeled using a bootstrap subsample of training data and a subsample of predictor variables. An averaged response is used to create the final test data prediction. A regression tree is formed by repetitive partitioning of variable data space such that the Residual Sum of Square (RSS) at each node is reduced. A bootstrap sample of data is obtained from training data with replacement^1^.

Residual Sum of Squares, RSS is given by:

2 $$\mathrm{RSS}=\sum_{c=1}^{n}\sum_{i=1}^{{n}_{c}}{({y}_{i}-{m}_{c})}^{2}$$

3 $${m}_{c}=\frac{1}{{n}_{c}}\sum_{i=1}^{{n}_{c}}{y}_{i}$$

*c* is the no. of nodes, ${n}_{c}$ is the no. of data points in a node, ${y}_{i}$ is the observed or actual response value.

To accomplish this, each node is split to reduce the RSS to the greatest. This is performed by contrasting several split possibilities utilizing various variables and split points within those variables. When a split is completed, two nodes are created, and then more splits are performed until the number of data points in each node reaches a predetermined limit^1^.

The hyper parameters used for the ML model are as given below:

1. The maximum no. of decision trees used for this model is 100.
2. The criterion used for this model is RMSE.
3. The maximum depth in a decision tree is allowed until purity is reached.
4. The maximum no. of splits set at each node is 6.

## Variable ranking

In this study, the variable ranking was used to show which variables significantly impact rate decline prediction and to rank them in order of priority. It can also be employed for eliminating the features but due to the availability of limited data, the entire dataset is utilized, and none of the features were removed. This data analysis was carried out to thoroughly understand the dataset before using it to make predictions.

Two algorithms were employed in this study to accomplish this:

- Classification using Chi Square Test (χ^2^).
- Regression using *F*-tests (fsrftest).

### Classification using Chi-Square Test (χ^2^)^8^

The Chi-Square Test is a statistical method that can solely be employed whenever the test statistic under the null hypothesis is chi-squared distributed. It is used to test the presence of a statistically significant difference between the expected and actual frequencies in one or more categories of a contingency table. Individual chi-square tests are performed to determine whether each parameter is independent of a response parameter^8^.

The following formula is employed when χ^2^ is utilized for testing the interdependencies between variables,

4 $$ \upchi ^{2}  = \frac{{(O - E)^{2} }}{E} $$

where, *O* represents the observed frequencies of the entries of the table, E represents the expected frequencies of the entries of the table.

### Regression using F-tests (fsrftest)^9^

The F-test is a statistical test that compares statistical models fitted to a data set to identify the model that best fits the population from which the data were sampled. It compares statistical models equipped with a data set to identify the model that best fits the population from which the data were sampled. It derives from evaluating a decomposition of the variability in a set of data in terms of sums of squares and is sensitive to non-normality^9^.

## Methodology

In this study, data was collected from the website of the Montana Board of Oil and Gas Conservation. The outlier wells, i.e., wells with higher or lower production or completion features, were removed. Only wells featuring a production history of more than 96 months (i.e., 8 years) were considered for this study. The predictor variables employed in the ML model is shown in Table 2.

**Table 2:** Description of the predictor variables employed in the ML model.

Sl. No.	Predictor variables	Description
1	Initial flow rate (${q}_{i}$)	Maximum flow rate
2	Proppant amount	Total amount of proppant utilized during hydraulic fracturing
3	Fracturing fluid amount	Total amount of fracturing fluid utilized during hydraulic fracturing
4	Stages	Total no. of fracturing stages
5	TVD heel-toe difference	TVD of well's heel minus TVD of well's toe
6	Completion length	Difference between the first and the last perforation
7	Measured depth (MD)	Actual depth of the hole drilled to any point along the wellbore
8	Total vertical depth (TVD)	The vertical depth from the surface to the depth of interest
9	Initial 24-h period oil produced	Oil produced during the initial 24-h period
10	Initial 24-h period gas produced	Gas produced during the initial 24-h period
11	Initial 24-h period GOR	GOR produced during the initial 24-h period
12	Tubing size	Size of the tubing used
13	Oil API gravity	API gravity of the oil produced
14	Flowing tubing pressure	Tubing pressure when the oil is flowing through the tubing
15	Bottom hole maximum treating pressure	Maximum pressure of the liquid inside the wellbore at the perforations at the bottom of a well that will fracture the rock
16	Bottom hole average treating pressure	Average pressure of the liquid inside the wellbore at the perforations at the bottom of a well that will fracture the rock
17	Average hydraulic horse power (HHP)	A measure of the energy per unit of time that is being expended across the bit nozzles

Cluster analysis is being used for exploratory analysis, in which well data is split into four clusters based on initial flow rate (qi) quartiles, (In this study, it's considered equivalent to the maximum flow rate), which was observed to be the most important predictor in this study as well as previous studies in a different shale region^1,8,9^. The primary objective of the exploratory analysis is to highlight trends and the relative importance of variables and include human judgement and machine learning algorithms to get more accurate conclusions. When the production rate decline for a large number of wells in a given oil field is required with limited production data, this method may be advantageous. Unlike commercial reservoir simulators, this method does not require precise knowledge of reservoir features like core data, well log data and other information that is sometimes inaccessible^1,8,9^.

The main objective of this study is to develop a ML based model that can be employed for the prediction of production rate decline for a large number of Bakken Shale wells in a very shorter period. This method will be much faster than the commercial reservoir simulators as it does not require solving a large number of finite difference equations.

**Figure 22:**
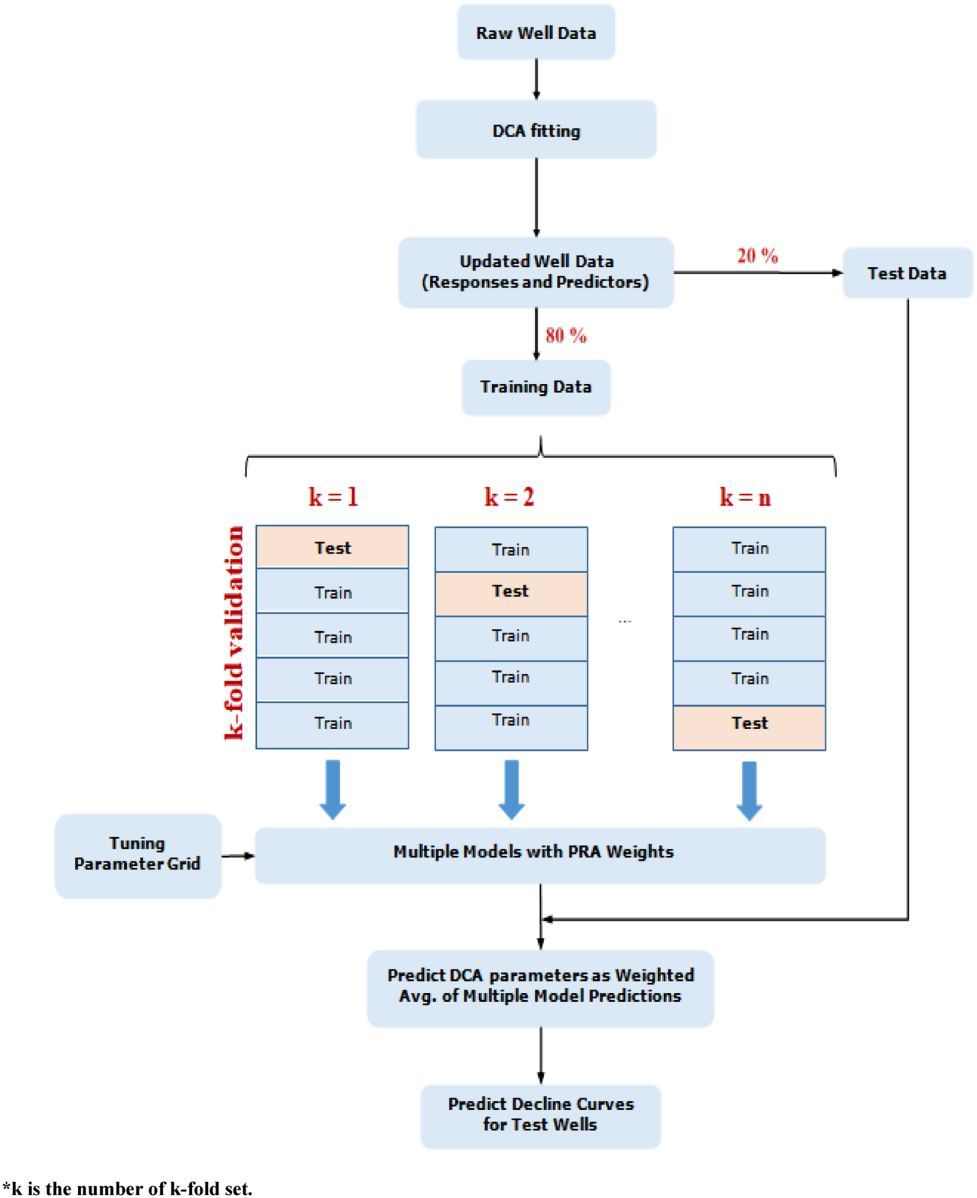
Workflow to build multiple models and average them (modified from Vyas et al.^1^).

The entire collected well data was used in machine learning-based predictions. The detailed prediction flow is shown in Fig. 22. Data were collected from 150 wells that had all the needed variable data available and also showed smooth decline in oil rates. A total of 120 wells were selected randomly as training wells, with the remaining 30 wells serving as test wells. Several of the available parameters were eliminated from the final dataset as their corresponding values were unavailable for all of the selected wells, indicating that no data was unavailable for any of the selected wells' parameters.

It has been observed that using the entire training dataset to develop a machine learning model can result in considerable errors owing to data variability. To overcome this limitation, an alternative approach used in this work, included Cross-Validation employing k-fold Validation and Model Averaging using the ensemble technique (Polyak–Ruppert averaging).

Steps involved in training the model:

1. The data is split into two categories: training and test.
2. Training data is further divided into 20 folds.
3. The model was evaluated in one of the folds while the other folds were used for training. Therefore, 20 folds result in 20 models.
4. At the end of the training, we shall have multiple machine learning models derived from a single set of training data by employing various samples of training data to minimize the problem of over fitting.
5. The tuning parameters for each model in a machine learning process would be distinct, resulting in various predictions for test data wells.
6. In each model training, the RF algorithm is used to get the optimized values (i.e., minimized error) of τ and n, as explained.
7. The weights of these models are determined using the Polyak–Ruppert averaging technique, which is based on test data error. The samples of training data indicated in step 1 corresponds to the test data here.
8. Finally, the weighted average of these models' responses predicts decline curve parameters (n and τ) and, as a result, decline curves for the test data wells.

## Limitations

1. In this study, only those wells are included that have a smooth production rate decline. Well with noisy data should not be used for this type of study.
2. The data for this study has been taken from Bakken Shale oil reservoir. If this methodology is applied to a new dataset belonging to a different field, the new ML model needs to be trained for the new dataset but the overall methodology should remain the same.
3. This study did not include various important parameters like porosity, permeability, reservoir pressure, etc. since corresponding data were not available.

This method necessitates the availability of a good dataset for all the parameters to measure the effect of all the possible parameters on the rate decline prediction. In the future, this study may be extended to apply the method developed to other shale oil reservoirs to test its applicability and accuracy.

## Data Availability

All data generated or analyzed during this study are included in this published article.

## Abbreviations

DCA: Decline curve analysis
EUR: Estimated ultimate recovery
GOR: Gas–oil ratio
MD: Measured depth
RF: Random forest
RMSE: Root mean squared error
RSS: Residual sum of squares
SCF: Standard cubic feet
SEDM: Stretched exponential decline model
TVD: Total vertical depth
LSSVM: Least square support vector machine
ANN: Artificial neural network
RMSE: Root mean square errors
